# The Economic Burden of Chronic Diseases with Co-Occurring Depression and Alcohol Use Disorder for People in the Western Cape, South Africa

**DOI:** 10.1016/j.ssmmh.2023.100268

**Published:** 2023-12

**Authors:** Vimbayi Mutyambizi-Mafunda, Bronwyn Myers, Katherine Sorsdahl, Amarech Obse, Crick Lund, Susan Cleary

**Affiliations:** 1 Health Economics Unit, School of Public Health, University of Cape Town, Cape Town, South Africa; 2 Curtin enAble Institute, Faculty of Health Sciences, Curtin University, Perth, WA, Australia; 3 Mental Health, Alcohol, Substance Use and Tobacco Research Unit, South African Medical Research Council, Francie van Zyl Drive, Tygerberg, Cape Town, 7505, South Africa; 4 Division of Addiction Psychiatry, Psychiatry and Mental Health, University of Cape Town, Cape Town, South Africa; 5 Alan J Flisher Centre for Public Mental Health, Department of Psychiatry and Mental Health, University of Cape Town, Cape Town, South Africa; 6 Centre for Global Mental Health, Health Service and Population Research Department, Institute of Psychiatry, Psychology and Neuroscience, King’s College, London, UK

**Keywords:** global mental health, depression, alcohol, HIV, diabetes, economic burden, LMIC^1^

## Abstract

There is limited evidence on the economic burden of chronic diseases with co-occurring depression and alcohol use disorder (AUD) for people in low-and middle-income countries. We describe patient costs related to the utilisation of services and identify factors associated with the economic burden of co-occurring depression and AUD for people with HIV and/or diabetes using government health services in South Africa. We used baseline data from participants enrolled in a cluster randomised controlled trial (RCT). The sample (N=1,340) comprised participants classified as having risk of depression but not AUD (*n*=689), risk of AUD but not of depression (*n*=221); or risk of depression and AUD (*n*=430). We measured total patient costs (direct patient costs (out-of-pocket payments (OOPP) plus indirect patient costs), and catastrophic costs. We applied a conceptual framework to guide multiple linear and logistic regression analyses examining factors associated with economic burden. Mean monthly total costs per patient and the percentage of these total costs attributable to OOPP were (US$9.78 [56.13%]; US$5.98 [24.58%]; US$7.16 [34.07%]) for the depression, AUD, and AUD and depression groups respectively. The depression group reported significantly more visits to private healthcare providers, higher OOPP and higher prevalence of catastrophe than other groups. OOPP were positively associated with urban location and higher educational attainment. Total patient costs were positively associated with urban location, HIV and diabetes comorbidity, and being employed. Higher utilisation was associated with greater odds of income loss. Results indicate a concerning economic burden in people with a chronic disease and co-occurring depression or AUD and suggest that cost and time may present barriers to accessing care. Given that psychological treatments for mental health conditions are largely unavailable in government health services, improving access to care for the most vulnerable may require coordination of financial risk protection mechanisms alongside scale-up of effective first-line psychological treatments.

## 1 Introduction

There is a large treatment gap for mental health conditions like depression and alcohol use disorder (AUD) in low- and middle-income countries (LMICs) such as South Africa (Docrat et al., 2019a; Jack et al., 2014). The economic consequences of these untreated conditions include out-of-pocket payments (OOPP) associated with treatment seeking and the economic costs of lost wages and lost productivity for the individual and society (Lund et al., 2013). International evidence suggests that the economic consequences of untreated depression and AUD are influenced by co-occurring chronic diseases (Chisholm et al., 2003; Simon et al., 2023). In South Africa, communities characterised by high levels of economic and social adversity (Klasen, 1997) have the highest prevalence of both chronic diseases (including HIV and diabetes) (Kim et al., 2021; Oni et al., 2015) and untreated depression (Cuadros et al., 2019; Gibbs, Govender & Jewkes, 2018) and AUD (Andersson et al., 2018; Marx et al., 2021; Probst et al., 2018). Further, a substantial proportion of people living with HIV and/or diabetes in these communities have undiagnosed depression and AUD (Kagee, 2010; Myers et al., 2021). The bidirectional relationship between economic adversity, depression, AUD and chronic diseases has been well-documented (Bhardwaj & Kohrt, 2020; Lee et al., 2023; Mendenhall et al., 2017; Mendenhall et al., 2022; Peprah et al., 2022; Singer & Clair, 2003). Economic and social adversity, shaped by the apartheid history of the country (Coovadia et al., 2009; Mayosi et al., 2012) are both risks for and consequences of (untreated) depression (Gibbs, Govender & Jewkes, 2018), AUD and chronic diseases. Depression and AUD impact on chronic disease care engagement and outcomes (Myers et al., 2021; Myers et al., 2022; Neuman et al., 2012; Truong et al., 2021), but these mental health conditions may also arise from the burden associated with having a chronic disease (Kagee, 2010). These interacting health conditions, and the clustering of these conditions in communities characterised by economic adversity have been termed syndemics (Singer & Clair, 2003). Despite evidence of a mental health and chronic disease syndemic in South Africa (Mendenhall et al., 2022; Workman, 2022), little is known about the economic burden for people with a chronic disease and co-occurring depression and/or AUD. In addition to this, the evidence base on patient costs and economic burden in LMICs is still concentrated around infectious diseases such as tuberculosis (TB) and human immunodeficiency virus (HIV) (Foster et al., 2015; Fuady et al., 2018; Prasanna et al., 2018) with limited but growing evidence for the costs associated with untreated depression and AUD (Christensen et al., 2020; Khan, 2016; Manthey et al., 2021; Ranaweera et al., 2018).

To inform policy on the financial risk protection mechanisms that may be required to mitigate this economic burden, more empirical evidence is needed around the costs incurred by people living with these co-occurring mental and physical health conditions and the adverse social factors that contribute to these costs. Adoption of appropriate financial risk protection mechanisms can improve access to health services and patient health outcomes (Lund et al., 2011), and support initiatives to integrate mental healthcare into primary care services in these high chronic disease burden contexts (Chuah et al., 2017; Ngo et al., 2013; Park et al., 2023; Patel et al., 2013; Thornicroft et al., 2019; UNAIDS, 2022). To address this information need, we use baseline data from a cluster randomised controlled trial (RCT) to describe pre-treatment patient costs among trial participants with HIV and/or Diabetes Mellitus (DM) and co-occurring depression and AUD within the Western Cape province of South Africa (Myers et al., 2022). We also explore factors associated with this economic burden in this cross-sectional sample.

## 2 Analtyical Framework

The economic burden of illness in LMIC settings is well documented and associated with the medical poverty trap (Foster et al., 2015; McIntyre, Thiede & Birch, 2009; McIntyre et al., 2006; Russell, 2004; Whitehead, Dahlgren & Evans, 2001). Economic costs of illness can lead to poverty which can be short term, associated with “health shocks” from acute conditions such as malaria (Chuma, Gilson & Molyneux, 2007; Mugisha et al., 2002; Russell, 2004), or long term related to ongoing “lumpy” health expenditures and asset losses with intergenerational fiscal consequences in chronic conditions like HIV (Foster et al., 2015; Russell, 2004). There is a limited but growing evidence base for the economic costs associated with mental health conditions (Cleary et al., 2020; Docrat et al., 2019a; Hailemichael et al., 2019a; Katana et al., 2020; Lund et al., 2019; Rajan et al., 2020). These economic costs include OOPP, categorised as the direct medical and related financial costs of care (WHO, 2015). In addition, people living with mental health conditions also incur indirect costs due to illness. These include income losses as people take time off work to access treatment, time lost from work when unable to work and lost productivity due to illness. The economic burden arises from these direct and indirect costs as the person seeks care and draws on social capital resources to mitigate the financial consequences of ill health (Russell, 2004).

**Fig. 1:**
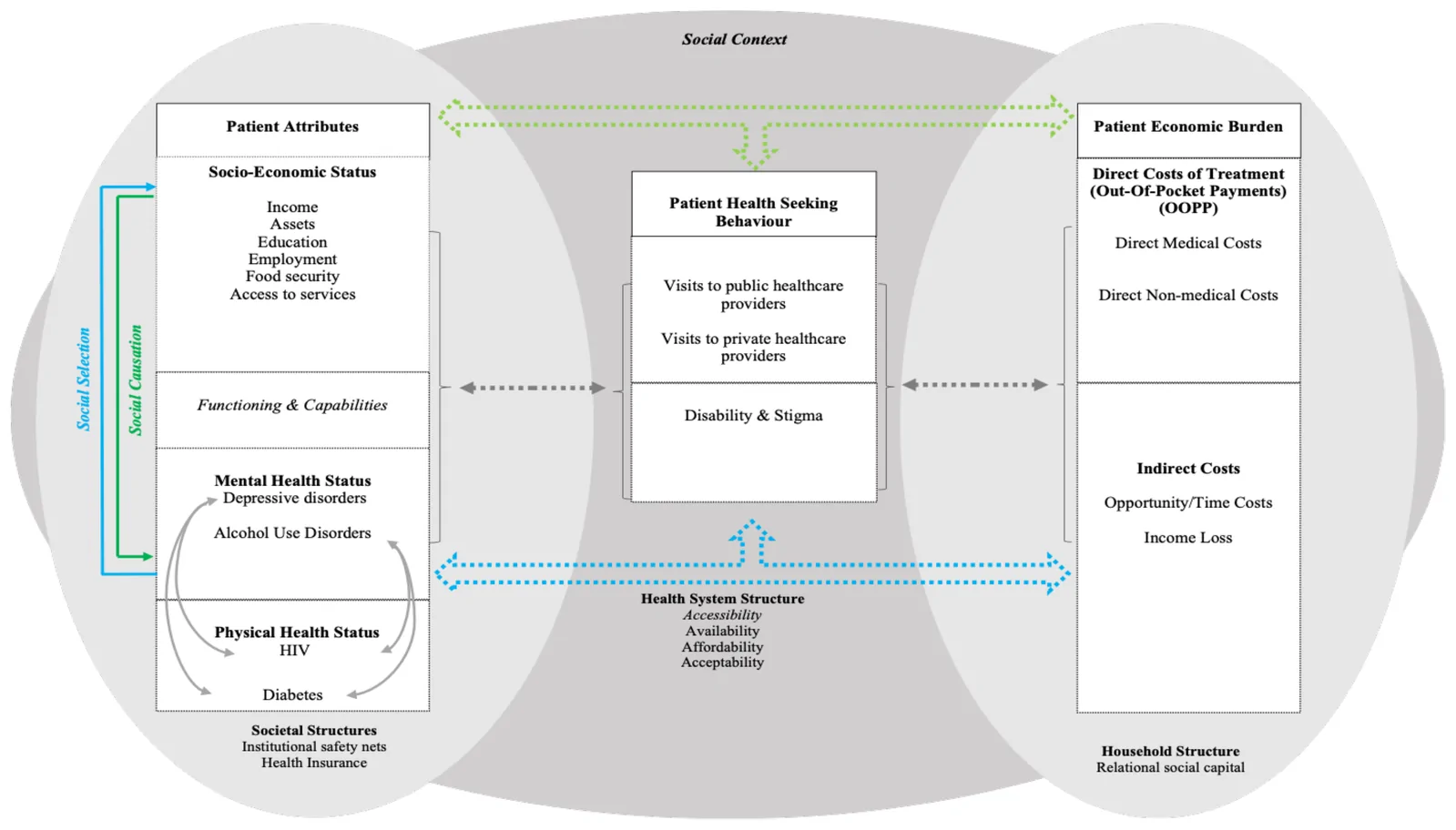
Analytical framework

Our conceptualisation of economic burden draws from previous frameworks linking the economic consequences of illness to a person’s treatment seeking behaviour as they interact with the health system (Foster et al., 2015; McIntyre et al., 2006; Russell, 2004), incorporating a person’s reduced ability to translate capabilities into income which is uniquely associated with mental health conditions (Lund et al., 2011). Our analytical framework is based on the social causation and social drift theories which have been used to conceptualise the relationships between mental health and economic outcomes (Lund et al., 2010). *Social causation* suggests that the difficulties and distress associated with circumstances of economic scarcity and instability can lead to mental distress, whilst *social drift* proposes that mental health conditions can result in more healthcare expenditures and lost income due to disability and stigma. Our framework (Figure 1) links an *individual’s attributes, health seeking behaviour* and *economic burden*. This micro-economic analysis of economic burden views an individual’s *socio-economic status* as arising from the combination of their income, assets, education, employment, food security and access to essential services (Booysen et al., 2008; Hailemichael et al., 2019b; Tirfessa et al., 2019). Socio-economic conditions can contribute to or provide a buffer against mental health problems. Socio-economic status has a bi-directional relationship with the *functionings* that allow a person to maximise their *capabilities* (Sen, 2005) and lead a productive and functional life (Lund et al., 2011). Mental health conditions affect socio-economic well-being both directly and indirectly by permeating an individual’s *functionings and capabilities*. These personal *attributes* (socio-economic and mental health status and associated functionings and capabilities) influence care seeking. *Health seeking behaviour* captures an individual’s interactions with the health system through the type and frequency of visits to different healthcare providers. A person’s mental health *disability* is intertwined with their physical *disability* (Prince et al., 2007).

For people living with mental health conditions in LMICs this physical disability is characterized by comorbidity with high burden chronic communicable and/or non-communicable conditions including HIV, DM, hypertension (Ngo et al., 2013), and more recently Coronavirus Disease 2019 (COVID-19) (Cénat et al., 2021; Kola et al., 2021). When these comorbidities are experienced in adverse social contexts, the resulting syndemic coupling (Peprah et al., 2022) may amplify economic burden. The non-disease social ecosystems that interact with biological comorbidities and influence care seeking are both structurally (*societal structures, health system structures and household structures*) and systemically embedded in the broad *social context*.

Care seeking is thus mediated by the presence of other chronic health conditions and adverse social conditions including disease related *stigma* (Anvari et al., 2022; Makhmud, Thornicroft & Gronholm, 2022; Monnapula-Mazabane, Babatunde & Petersen, 2022). Interactions with the health system have consequences that impact on a person’s economic well-being. The *economic burden* of mental illness arises from treatment costs, productivity and income losses due to the disability and stigma associated with mental illness (Lund et al., 2013) and multi-morbidities (Karan et al., 2022; La et al., 2022; Lewis et al., 2022; Sum et al., 2018; Tran et al., 2022). Individual attributes are interlinked with *societal structures* including access to health insurance, disability grants, and financial services. Health seeking behaviour is experienced in the context of the *health system structures* determining the accessibility, availability, and affordability of services (McIntyre, Thiede & Birch, 2009). Household economic burdens/coping strategies have an impact on the individual’s financial circumstances and their mental health, thus economic challenges of ill health are managed in the context of *household structures*, incorporating their relational capital resources. This framework hypothesizes that an individual’s mental health and socio-economic status contribute to their functionings and capabilities, and influences health seeking behaviour, modified by disability and disease-related stigmas. Together these factors contribute to the economic burden experienced by the individual, within their societal, health system and household contexts.

Through this cross-sectional analysis of individual level economic costs of illness, we aim to contribute to the empirical evidence on the social causation and social drift theories by examining factors contributing to economic burden and assessing associations between total costs of illness and predictors highlighted in the analytical framework (Figure 1 & Box 1).

## 3 Method

### 3.1 Study design

The study analyses baseline data from “Project MIND”, a three arm cluster RCT of different approaches to non-specialist health worker (NSHW)-delivered psychological interventions for people treated for a chronic disease in government primary health care (PHC) clinics in the Western Cape Province of South Africa (Myers et al., 2022; Myers, Bronwyn et al., 2018). Trial participants were clinic attendees receiving treatment for HIV and/or DM who screened positive for depression and/or AUD. Study assessors used computer-assisted personal interviews to collect self-reported data on participant demographics, socio-economic and health status at baseline, 6- and 12-month assessments.

### 3.2 Setting

#### 3.2.1 Social setting

South Africa has a dual health system with access to healthcare services determined by ability to pay (Ataguba & McIntyre, 2012; McIntyre, 2012). Government primary care facilities typically serve low-income patients as there is no payment for these healthcare services, whilst higher income patients access private providers by paying out-of-pocket at the point of service or through private health insurance (James et al., 2018). In line with the country’s history of racial segregation which extended to health services (Price, 1986) most government primary care facilities are located in low-income areas and still largely serve people of colour (Mhlanga & Garidzirai, 2020). Low-income communities face multiple social deprivations including limited access to sustainable housing, municipal services, quality education, affordable, reliable and safe transport, safe recreation, and policing (Klasen, 1997). In addition to this many of these communities are plagued by high levels of unemployment, food insecurity, gangsterism, violence and substance abuse. The high costs of fresh nutrient rich foods, long commutes to work from marginalised living areas limiting time for food preparation in largely single parent households, coupled with access to cheap calorie dense foods has promoted obesogenic environments in these communities (Battersby et al., 2023). The careful marginalisation and deprivation of people of colour during apartheid laid the foundation for intergenerational deprivation and left indelible scars of poverty, inequality, and general social adversity which are evident in these populations (Klasen, 1997). These vulnerable segments of the population are therefore at the interface between several comorbidities and circumstances of social adversity.

#### 3.2.2 Study setting

Study sites were government PHC clinics identified through collaboration with the Western Cape Department of Health (WCDOH). The WCDOH purposively selected the clinics to meet trial requirements for co-location of HIV and DM treatment services. Selected facilities were a fair representation of the facilities in the province varying in size and extent of primary care services offered. These PHC facilities represented a diverse range of urban/peri-urban (15) and rural (9) settings across most health districts in the province (Myers et al., 2022) serving a population of 8 million people (Boulle et al., 2019). These facilities operate in a government health service plagued with shortages of mental health care providers (NDoH, 2023), limited capacity of other providers to deliver mental health treatments (Jacobs et al., 2021) and chronic underfunding (Docrat et al., 2019b). These systemic problems and other factors have resulted in mental health treatments being largely unavailable in these facilities (Myers et al., 2022). This is despite widely endorsed recommendations (Funk et al., 2004; Mahomed, Asmall & Freeman, 2014; Sorsdahl et al., 2021), strategic policy frameworks (NDoH, 2013) and primary care treatment guidelines (NDoH, 2016) promoting progressive, stepped care approaches to the treatment of mental health conditions in primary care settings (Myers et al., 2022; WHO/UNHCR, 2015).

### 3.3 Sample size and participants

Eligible participants were adults (18 years and older) taking medication for DM and/or antiretroviral therapy (ART) for HIV who screened positive for AUD and/or depression. Screening for AUD was done using the 10-item Alcohol Use Disorder Identification Test (AUDIT) (Babor et al., 2001); participants with an AUDIT score ≥8 were enrolled in the study. Participants who scored ≥16 on the Centre for Epidemiological Studies on Depression Scale (CES-D) (Radloff, 1977) were also eligible. Participants who reported using pharmacotherapy or receiving counselling or other services for a mental health concern were excluded from study participation (Myers et al., 2022). More details about the recruitment and data collection procedures are set out in the trial protocol (Myers et al., 2018b). The final realised analytical sample for this study was *N*=1340 (Myers et al., 2022).

### 3.4 Measures

South Africa has a highly pluralistic healthcare system. Government PHC services are free at the point of use, while outpatient or emergency department visits and inpatient stays at government hospitals incur variable fees depending on household income. Beyond these government services, a range of private providers can be used on a fee for service basis and a variety of medicines and special foods can be purchased at private pharmacies and shops. We designed a bespoke questionnaire to capture the utilisation of these government and private services and associated costs, using a 4-week recall period for costs related to ambulatory care and a 6-month recall for costs related to hospital care. While most of the costs were collected on a ‘per visit basis’ (e.g., “how much did you spend on transport to access the clinic today?”), we also collected data on the frequency of service utilisation across the range of government and private services. We then used these data to transform the ‘per visit’ estimates into average monthly costs. Costs were collected in South African Rands and were converted to United States Dollars (US$) using a 2017 (year of baseline assessment) exchange rate of US$1: R12.89 (www.Oanda.com).

We categorised costs as direct medical OOPP, direct non-medical OOPP and indirect costs, all expressed on a monthly basis in US$. Direct OOPP included medical and related non-medical costs paid out-of-pocket (OOP) in accessing government and private healthcare services. Medical costs consisted of any fees incurred for consultations and diagnostic procedures, including traditional or faith-based consultations. Medical costs also included the costs of health care products including pharmaceuticals, traditional and faith-based medicines and any costs related to special diets associated with treatment for the chronic health condition. Non-medical costs included transport to and from facilities and any subsistence (food and accommodation) costs.

Indirect costs were estimated from the opportunity costs of an individual’s time associated with a clinic visit and income losses. Opportunity costs were valued using the equality of wages approach which entails valuing time according to the mean income across the group (WHO, 2015). This approach was preferred to the minimum wage approach which may overstate time costs in contexts of high unemployment (Cleary et al., 2013; Foster et al., 2015; Pillai et al., 2019). Income losses were valued as the lost income reported by the participant related to their routine HIV/DM clinic visit, and over the preceding 4-week recall period. Total costs were estimated as the sum of direct and indirect costs. Binary indicator variables were generated to examine health-related catastrophe using thresholds of health-related OOP expenditures ≥ 10.00%, 20.00%, 30.00%, 40.00% and 50.00% of patient income (Barter et al., 2012; Hailemichael et al., 2019a). A binary indicator variable was also generated to capture reported income losses.

Socio-economic and demographic explanatory variables include participant age, sex, race, marital status, educational attainment, employment status, clinic location (proxy for residential location), housing stability, socio-economic status, and food security of household members (Table 1). Due to the multi-dimensionality of poverty in low-income settings and the categorical nature of variables capturing poverty, a composite index of socio-economic status was generated using multiple correspondence analysis (MCA) (Abdi & Valentin, 2007; Booysen et al., 2008).

Health status explanatory variables included mental health status captured by CES-D and AUDIT scores, whilst HIV and DM measures indicated chronic disease status, and general health and well-being was captured using an index of Health-Related Quality of Life (HRQoL) (Table1). Dichotomous variables were generated for the chronic disease (HIV and/or DM) and mental health (depression and/or AUD) variables. Cut-offs used to determine mental health status were CES-D≥16 for risk of depression (Radloff, 1977). AUD risk was considered present when AUDIT score≥8 (Babor et al., 2001). A composite index of Health-Related Quality of Life (HRQoL) was generated using the EQ-5D-3L (capturing mood, anxiety and functional ability) and valued using the UK time trade-off value set (Rabin & de Charro, 2001). This index can take a maximum possible value of 1 reflecting perfect health and minimum value of 0. A dichotomous variable was generated for HIV/DM-related stigma, based on single-item questions on stigma-related barriers for HIV and DM medication adherence (Saberi et al., 2015).

### 3.5 Data analysis

Data were analysed using Stata 15.1 (Stata Corp. 2015), using Stata’s survey analysis platform to account for clustering. Descriptive statistics (means and standard errors) on participant demographic, socio-economic and health status were calculated for the MIND population and across combinations of depression and/or AUD. These mental health condition categories (depression only, AUD only, and depression & AUD) were further applied to the comparative descriptive statistics on health service utilisation and patient costs. As a result of the uneven distributions of cost data, mean values were presented as measures of central tendency, with standard errors. Standard deviation (SD) and inter-quartile ranges (IQR) were reported for income variables. We tested for significant differences in utilisation, cost, and other variables across the three categories of mental health using Kruskal-Wallis tests. We used the Chi-squared test to test for significant differences in proportions of participants incurring catastrophic costs across mental health categories.

We used regression analysis to examine the relationship between the factors associated with the economic burden of mental illness as per our conceptual framework. To examine these relationships, we hypothesize that the economic burden is captured by OOPP and total costs (TC) comprising of OOPP and indirect costs (IC) as the continuous dependent variables in our multiple linear regressions (Box 1.) Explanatory variables included: an index of socio-economic status; HRQoL index; CES-D score; AUDIT score; total ambulatory visits; hospital inpatient days; chronic disease (HIV and/or DM) categorical variables; and an indicator variable for HIV/DM-related stigma. We controlled for age, sex, race, marital status, employment, education, food security, and location. Variables were omitted from the regressions if multicollinearity was detected. For the multivariate regression analyses the mental health explanatory variables were applied in their continuous form i.e., using the CES-D and AUDIT scores, allowing for interpretation according to linear variations in symptom severity. To further explore predictors or drivers of economic burden we used previously described categorical indicator variables for health-related catastrophe and participant-reported income loss as the dependent variables in logistic regressions. Mental health status explanatory variables were used in their categorical form in the logistic regressions.

### 3.6 Ethics

Ethical approval for this study was granted from the South African Medical Research Council (EC 004-2/2015), the University of Cape Town (089/2015; 005/2017; and 186/2019), and Oxford University (OxTREC 2-17). The WCDOH also provided approval (WC2016_RP6_9) for the study to be conducted in identified facilities. Participants gave written informed consent for study participation.

## 4 Results

### 4.1 Descriptive characteristics

The overall sample comprised N=1340 participants, and sample sizes for mental health categories were depression only (DEP: *n*=689); AUD only (AUD: *n*=221); and depression and AUD (DEP & AUD: *n*= 430). Characteristics of the study population are presented in Table 1.

The majority of participants were female (76.04%), with the highest proportion of men in the AUD only group. The mean age was 45.50 years, with the highest mean age in the depression only group (49.64 years). Educational attainment was low with 87.54% of participants reporting some high school or less as their highest level of education. Unemployment was high (54.70%), and the depression and AUD group reported significantly higher levels of unemployment (64.88%) compared to the other groups (47.96%-50.51%), (Table 1). Almost 30.00% of participants reported that government grants were their main source of income and 60.00% of participants reported non-productive income (government grants, pensions, maintenance, remittances from family members) as their main source of income. The median monthly income was US$108.07 (Table 1), with income differences observed among the mental health groups (*p*=0.00). Approximately 7.00% of those employed had an estimated monthly income below the lower bound of the national food poverty line (StatsSA, 2020). Almost half (48.88%) the participants reported that members of their household were often or sometimes hungry (Table 1). Among participants with depression only, the majority had DM as their only co-occurring chronic disease (57.62%), whilst most participants with depression and AUD were living with HIV as their only chronic disease (77.21%) (Table 1). The HRQoL index indicated relatively lower health status for those with depression only with estimates of 0.76, 0.92 and 0.78 (*p*= 0.00) for the depression only, AUD only, and depression & AUD groups respectively (Table 1).

### 4.2 Health service use

On average, participants made 0.73 visits per month to the study clinic for their HIV and/or DM care, with no significant difference between mental health condition groups (Table 2).

Participants made an average of 1.11 ambulatory visits per month over and above their clinic visit for routine HIV/DM care. Those in the depression and depression and AUD groups had slightly more frequent visits on average with 1.19 and 1.18 visits per month respectively, compared to 0.70 visits for the AUD only group (*p*<0.05; Table 2). These ambulatory visits were driven by private provider consultations (suggesting participants were seeking additional care for complications, other comorbidities, or undiagnosed conditions). The number of private provider consultations differed significantly between groups (*p*<0.05) (Table 2). Across the study population, 7.23% of participants reported a hospital inpatient stay over the 6-month recall period, with a slightly higher proportion of participants with DM (8.34%) reporting a hospital stay compared to those with HIV (7.24%; *p*= 0.00). No significant difference was found across mental health condition groups.

### 4.3 Direct costs (OOPP)

Total OOP payments for the overall MIND population were US$3.86 per patient per month, with significant differences across mental health condition categories (*p<*0.05; Table 2). The depression only group incurred US$5.49 per month in OOPP (42.22% more costs compared to the MIND population average). The AUD only group incurred the lowest OOPP, US$1.47 (61.91% less than the MIND average). Total direct costs (OOPP) were highest in the depression only group, this was possibly driven by the OOP spend on private provider consultations (p<0.05) and special diets by these participants living with chronic disease and co-occurring depression (*p*<0.05; Table 2). Monthly spend on healthcare products, most notably spend on special diets, ranged from US$1.72 to US$ 0.09 across the mental health condition groups (*p*< 0.05). In the depression only group, the spend on special diets represented 31.32% of all out-of-pocket expenditures (Table 2). When we excluded special diets from total OOPP the depression patients continued to incur significantly higher total OOPP compared to the AUD only and the depression plus AUD groups (Table 2). Patients with AUD and co-occurring DM or co-occurring HIV and DM did not report any expenditures on special diets (Table 2).

### 4.4 Indirect and total participant costs

Total indirect costs (opportunity costs of time and income losses) were higher than direct costs in all groups apart from the depression only group (US$4.29 vs US$5.49; Table 2). When special diets were excluded from total OOPP, total indirect costs were higher than the latter across all mental health groups (Table 2). Total patient costs (direct OOPP and indirect costs) for the overall MIND population were US$8.32 per participant per month (US$7.30 excluding special diets) with the depression only group incurring US$9.78 per month, significantly more than the AUD only group (US$5.98). Mean monthly total costs per participant and direct costs as a percentage of total costs were: (US$9.78 [56.13%]; US$5.98 [24.58%]; US$7.16 [34.07%]) (including special diets) and US$8.07 [46.84%]; US$5.90 [23.55%]; US$6.79 [30.48%] (excluding special diets) for the depression only, AUD only, and AUD and depression groups respectively (Table 2).

### 4.5 Catastrophic costs

To further explore the economic burden of co-occurring mental health and physical health concerns we examine the relationship between participant healthcare costs and income. At the 10.00% threshold, 8.81% of patients in the overall MIND population incurred catastrophic OOPP (including special diets), compared to 11.76% and 4.52% in the depression and AUD groups (Figure 2a). When total costs were considered, 21.42% of the overall sample incurred catastrophic total costs (Figure 2a). Despite the depression group reflecting a relatively higher prevalence of catastrophic total costs than the other groups (23.22%), this difference was not significant (Figure 2a). Findings on depression were not significant in the regressions (Table 4). Extending the catastrophic cost analysis (OOPP including special diets) across a range of thresholds showed significant differences in the percentage of patients incurring catastrophe for all but the 50.00% threshold (Figure 2b). At the 40.00% threshold, the highest proportion of patients incurring catastrophe was in the depression group (2.18%) (Figure 2b). When special diets were excluded from OOP spend, statistically significant differences in proportions of patients incurring catastrophe where evident only at the 20% threshold (Figure 2b). The depression only group reflected the highest proportion of patients (4.21%) incurring catastrophe at the 20.00% threshold (Figure 2b).

### 4.6 Predictors of economic burden

Examining the predictors of OOPP (including special diets) using multiple linear regression (Table 3) indicates that participants in urban settings incurred higher OOP expenditures than rural participants (beta coefficient [β]=34.33 [95% confidence interval (CI)=9.09 – 59.56], *p*=0.01). Higher educational attainment (β =40.52 [95% CI= 5.38 – 75.65], *p*=0.02) also had a positive association with higher OOPP. A higher AUDIT score was associated with lower OOPP (β=-1.06 [95% CI= -2.10 – (-)0.03], *p*=0.04). Higher HRQoL was also associated with lower OOPP (β =-36.95 [95% CI= - 71.33 – (-)2.56], *p*=0.03). When the analysis was conducted with OOPP excluding special diets, a similar pattern of findings emerged, except for the AUDIT score which was no longer associated with OOPP.

When the analysis was extended to total patient costs (OOPP and indirect costs [opportunity costs of time and income losses]), urban location (β =54.23 [95% CI= 14.36 – 94.10], *p*=0.01), age (31-39 years) (β =38.88 [95% CI= 0.68 – 77.07], *p*=0.04), and being employed (β =64.87 [95% CI= 20.74 – 109.00], *p*=0.00), were positively associated with total costs (Table 3). Living with both HIV and DM was also associated with higher total costs (β =98.08 [95% CI= 12.05 – 184.10], *p*=0.02) (Table 3). When the analysis was conducted with special diets excluded from the total costs, similar significant associations were found, except for the age (31-39 years) coefficient which was insignificant.

A total of 93 (7%) participants in the study reported losing income due to sickness and treatment seeking. Further analysis (Table 4) of these drivers of economic burden revealed that being of Black African ethnicity (odds ratio [OR]=0.50 [95% CI=0.28 – 0.87], *p*=0.01) and being in the higher socio-economic group were associated with decreased odds of incurring income loss (OR=0.43 [95% CI= 0.27 – 0.69], *p*=0.00). Being employed was associated with an increased likelihood of incurring income loss (OR= 29.38 [95% CI= (11.06 – 78.00)], *p*=0.00). Increased ambulatory visits were associated with an increased likelihood of incurring income loss (OR=1.04 [95% CI= (1.00 – 1.07)], *p*=0.01) (Table 4).

High risk of AUD (OR=0.48 [95% CI=0.30 – 0.78], *p*=0.00) and higher HRQoL (OR= 0.38 [95% CI= 0.18 – 0.80], *p*=0.01) were both associated with lower odds of incurring catastrophic OOPP (Table 4). Living with DM as the only chronic comorbidity (OR= 1.87 [95% CI= 1.05 – 3.33], *p*=0.03), being of Black African ethnicity (OR= 1.83 [95% CI= 1.03 – 3.23], *p*=0.03) and increased ambulatory visits to healthcare providers (OR= 1.04 [95% CI= 1.00 – 1.08], *p*=0.04) were associated with higher odds of incurring catastrophic OOPP. When the analysis was conducted with OOPP excluding special diets, increased hospital visits and experiencing HIV/DM-related stigma were associated with increased odds of incurring catastrophic OOPP. HIV/DM-related stigma and living with both HIV and DM were associated with higher odds of incurring catastrophic total costs irrespective of whether special diets were included or excluded (Table 4).

## 5 Discussion

Our study found that OOP expenditures present a substantial economic burden to participants with a chronic disease and co-occurring depression and/or AUD. Total OOPP for the overall MIND population were US$3.86 per participant per month. Given high unemployment levels and that the majority of participants reported non-productive main sources of income, these OOPP constitute an economic burden for this group. Although government health services are free at the point of use, transport costs may be a financial barrier to accessing care. Participants in this study were only included if they were not receiving mental health treatments (Myers et al., 2022). It can be expected that transport costs may be a barrier to patient uptake of face-to-face consultations for psychological treatments should these WHO recommended (WHO/UNHCR, 2015) interventions be implemented in practice. Transport costs together with other costs of health seeking have been shown to be amplified in contexts where there are mental health and physical health syndemics and socio-economic inequality (Mendenhall et al., 2017). Evidence on patient costs for other high burden conditions in three LMIC settings suggests that financial risk protection through the provision of free treatment and diagnosis may not be enough to ensure equitable access to care for the most socio-economically vulnerable people (Mauch et al., 2013).

We also found that spend on other health services (public clinics other than the usual clinic visit for routine HIV and/or DM care and private healthcare providers) as well as expenditure on health care products are a significant contributor to OOPP, especially for those in the depression group. The spend on special diets by those living with depression (Table 2) is noteworthy. Further micro-analysis of these dietary costs by chronic condition reveals significantly higher spend on special diets for the DM sub-group across both the depression and the depression & AUD groups relative to the AUD only group (Table 2). This suggests that living with DM and depression may present a particular economic burden. This is in line with well-established literature on the association between depression and DM comorbidity (Egede & Ellis, 2010; Katon, 2008). People living with this comorbidity have been found to have excess healthcare expenditures in comparison to those without (Ciechanowski, Katon & Russo, 2000; Egede, Zheng & Simpson, 2002; Katon et al., 2008; Simon, Gregory E. et al., 2005). Similar findings have been noted in an LMIC context where a DM-depression centred syndemic has been observed (Mendenhall et al., 2017) and in low-income contexts characterized by similar socio-economic adversities (Weaver & Mendenhall, 2014). Literature on the dietary costs of people living with depression and DM in LMIC settings is limited with most reporting on macro-level OOP payments (Ankonu, 2019; Dipnall et al., 2015; Gemeay et al., 2015; Seuring, Archangelidi & Suhrcke, 2015), suggesting a need for more micro-economic analyses (Akena et al., 2015; Wallace et al., 2018). Grounding future economic analyses within a syndemics framework (Tsai & Venkataramani, 2016) may provide a more holistic view of costs and inform more appropriately targeted financial interventions. Just as patients do not experience multiple biological conditions within their bodies in silos, they may also not experience the associated costs of treatment for these conditions in silos.

The significantly lower OOP payments incurred by participants with AUD compared to those with depression suggests a possible healthcare self-neglect and crowding out of healthcare spending by this group perhaps in favour of alcoholic beverages. This is not surprising given that people with AUD in similar communities report spending almost a third of their household income on alcohol (Belus et al., 2022). In recent qualitative research in Ethiopia, individuals reported less money wasted, increased capacity to work and increased earnings due to the reductions in alcohol consumption associated with a brief AUD intervention (Zewdu et al., 2022). The syndemic coupling (Peprah et al., 2022) identified in African countries between substance use disorders and deleterious socio-political and economic circumstances suggests that interventions to improve access to treatment may be more effective if a synergistic approach that incorporates targeting both social and biological vulnerabilities is taken (Peprah et al., 2022). Improving access to effective treatments may reduce spending on alcoholic beverages which may in turn improve outcomes for comorbidities such as HIV and diabetes, and thus reduce the economic burden experienced by patients.

Our evidence also suggests that indirect costs contribute to the economic burden carried by participants. In LMIC settings where formal employment levels are low (the national unemployment rate in South Africa in 2017 was 27.70% (StatsSA, 2018)), income losses though difficult to quantify have significant consequences for livelihoods due to limited government provided economic safety nets (Govender et al., 2015). We found that higher socio-economic status may act as a buffer against health-related income loss. This points to the need for greater financial protection of low SES populations as these buffers are developed over time (even generations). Living with HIV as the only chronic comorbidity was associated with lower total and catastrophic costs suggesting that existing policies around HIV may be protective against the economic effects of ill-health. South Africa continues to have the largest HIV treatment program in the world (Bello et al., 2021; Mayosi et al., 2012; Meyer-Rath et al., 2017), with comprehensive services provided by government primary health clinics. This intensity of care for people with HIV may account for the protective effects that are coming through in this analysis. Financial safety nets provided by the state need to be further extended to people living with chronic diseases and mental health comorbidities, particularly those who are vulnerable to social drift.

We found that some participants incurred catastrophic OOPP at thresholds as high as 40.00% and 50.00% of income. Although this may appear to affect a relatively small proportion of patients, such expenditures magnify socio-economic insecurity and add further distress to people who are already severely economically vulnerable. We found that 21.42% of participants incurred catastrophic total costs using a threshold of 10.00% of participant income. A previous study on patient costs for TB/HIV in a similar setting showed between 53.00% and 67.00% of that sample incurred catastrophe at the 10.00% threshold based on total costs, this was driven mainly by income losses from job losses (Mudzengi et al., 2017). This suggests that even in low employment settings, the indirect costs of illness due to job losses can be a substantial contributor to income loss. This however may also be indicative of social causation, i.e., job loss and related income loss may contribute to worse depression. Our estimation of income losses was limited to income losses reported by participants associated with routine clinic visits and not going to work due to illness (4-week recall). The inclusion of income losses from job losses in the estimation of total income losses may result in even higher levels of people living with depression/AUD and co-occurring chronic disease experiencing catastrophic total costs.

## 6 Limitations

This analysis was limited to the variables and population included in the Project MIND trial. The trial only recruited participants with HIV and/or DM who screened positive for depression or AUD. It was therefore not possible to assess whether depression or AUD conferred an additional economic burden on patients with HIV/DM. Trial recruitment and eligibility may have impacted the sample resulting in older and sicker participants in the DM cohort. There was no distinction made between Type I and Type II diabetes patients at recruitment, therefore it was not possible to examine differences in costs by type of diabetes. In the project MIND RCT, mental health stigma and barriers to mental health treatment were not examined, primarily because all participants had agreed to participate in the trial. This exclusion limited the extent to which we could examine the effects of mental health stigma on economic burden. The relatively lower HRQoL score for participants with depression only and relatively higher scores for participants with AUD could be correct proxies for HRQoL or may be an indication that the instrument is not sensitive to AUD.

## 7 Conclusion and Recommendations

Overall, the evidence suggests a concerning economic burden for people living with HIV/DM and co-occurring depression/AUD. The indication is that a significant number of participants incurred catastrophic costs, when using either OOPP or total costs. Patient costs were higher in employed, working age participants residing in urban areas suggesting that cost and time may present as barriers to accessing treatment in those who are relatively less well-off and living in rural areas. This also suggests that for those who are spending on healthcare, these expenditures may be limiting investments in education and housing which improve long term socio-economic status. This also suggests that although access to public primary healthcare services in South Africa is free at the point of service, the health-related financial safety nets available in the form of free services and disability grants are not comprehensive enough to protect those with multi-morbidities and vulnerable socio-economic circumstances.

### 7.1 Recommendations for future research

Given these results we recommend the following potential avenues for future research. First, further application of the conceptual framework is required in longitudinal research to explore bi-directional pathways of causality. More evidence is needed to examine social causation, for example whether economic stressors like job and income loss contribute to worse depression; and social drift, for example whether worse depression and disability lead to more productivity and income losses over time. We suggest that these analyses of social drift and social causation are focused on mental health and chronic disease combinations which present especially harmful economic consequences as they interact with social adversity. Including a comparison population with no or less socio-economic adversity and similar co-occurrence of mental health and physical conditions in these analyses may provide further insights into economic impact. Second, micro-analyses of the economic cost of dietary patterns of people with DM and co-occurring mental health conditions in contexts of socio-economic deprivation are needed to complement the macro-level analyses available in LMICs. Third, further research is needed on the economic costs of AUD and chronic disease in socio-economically vulnerable populations. Fourth, household research is needed to explore coping mechanisms used to manage the economic burden associated with co-occurring mental and physical health conditions. Finally, mental health conditions and chronic diseases co-occur with particularly deleterious consequences in adverse social contexts, further research incorporating syndemic theory and methods (Tsai & Venkataramani, 2016) in analyses of economic burden is needed. This may provide a more holistic understanding of economic costs and inform more nuanced financial and health system interventions to relieve the burden carried by the most vulnerable.

### 7.2 Recommendations for policy

We recommend that policy makers in the Department of Health work with funders in ministries of finance to institutionalise financial safety nets for people living with depression/AUD and chronic disease to help with excess economic burden arising from transport costs, and healthcare products, in adverse social settings. The results of this study also suggest an income support policy will be protective of health-related catastrophic costs. The results indicate that these financial interventions are imperative for the scaling up of first line psychological treatments as participants in our study were already struggling with the OOP costs for accessing health services. We also recommend that public mental health researchers examine the socio-economic vulnerabilities and other factors that may present barriers to care for AUD and work with policy makers to examine the need for scale up of first line treatments for AUDs.

## Figures and Tables

**Fig. 2a:**
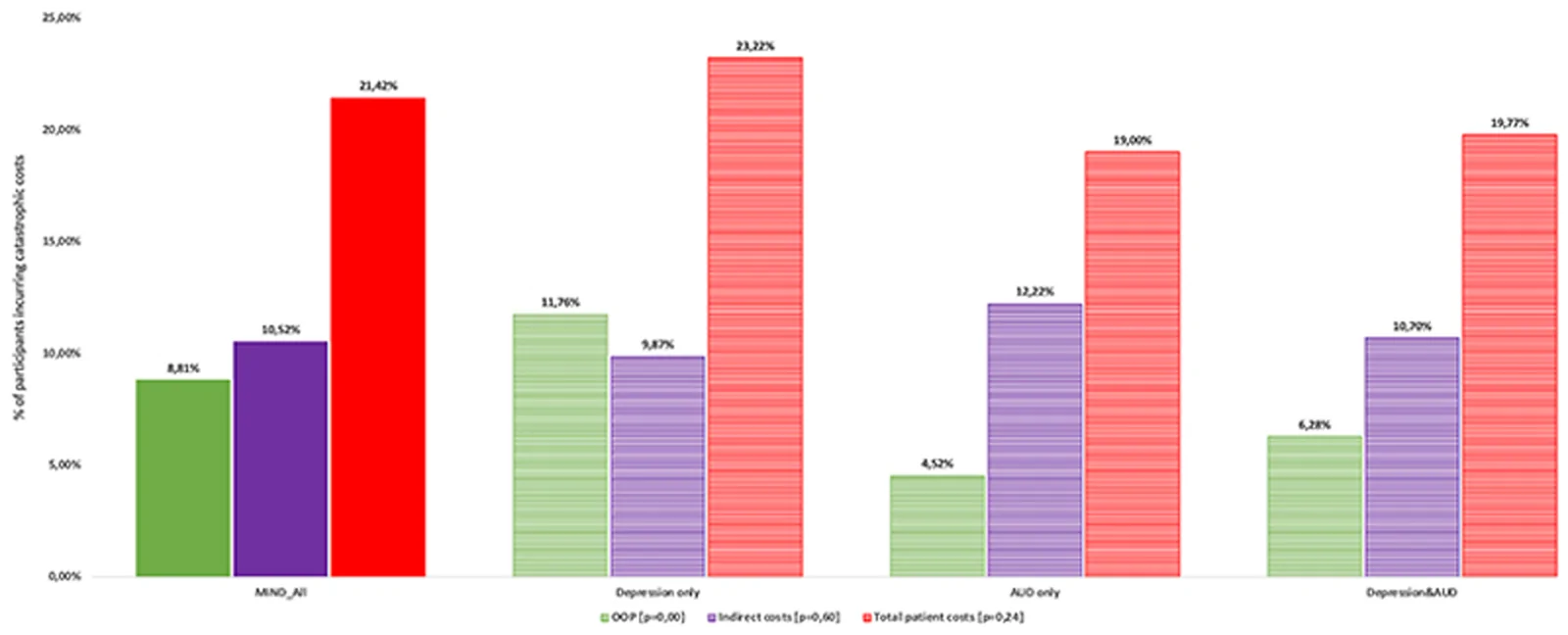
Percentage of participants incurring catastrophic costs by mental health condition

**Fig. 2b:**
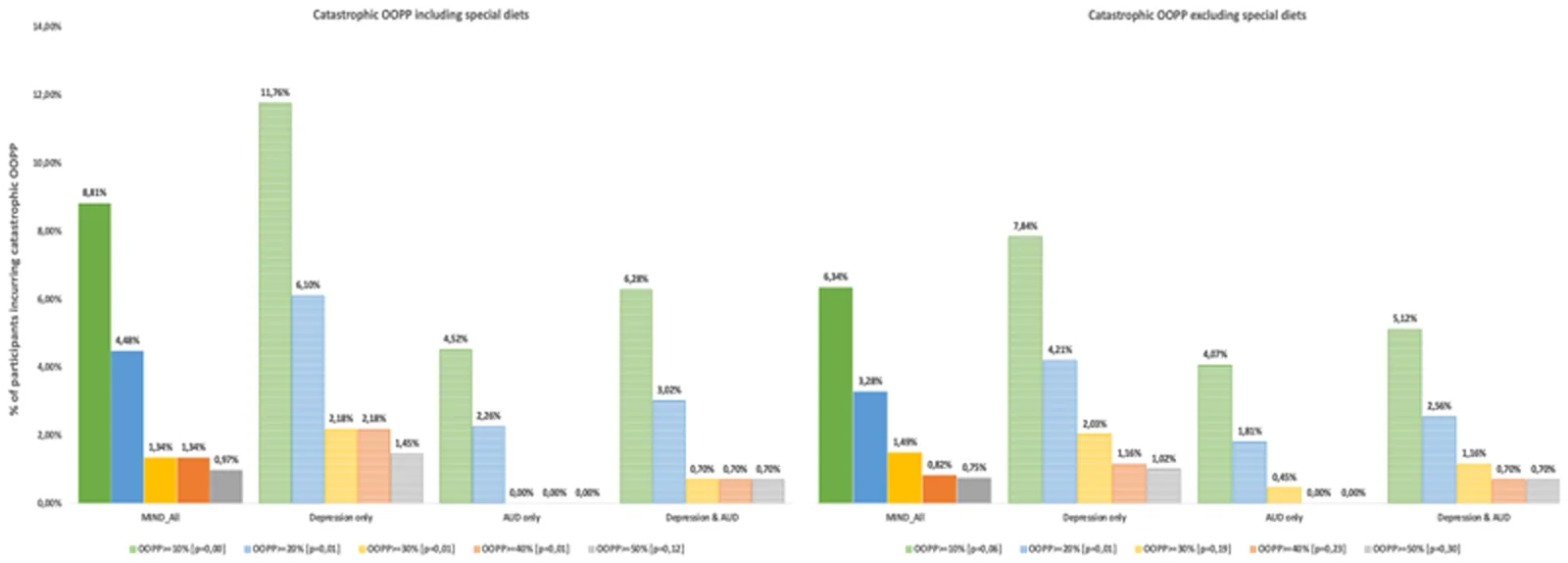
Percentage of participants incurring catastrophic OOPP by mental health condition

**Table 1:** Socio-demographic, socio-economic and health status indicators by mental health condition

	MIND (All)		Depression only		AUD only		Depression & AUD	
Variable type	(N=1340)		(N=689)		(N=221)		(N=430)	
**Demographic**								
Sex: female (n,%)	1019.00	76.04	586.00	85.05	118.00	53.39	315.00	73.26
Age [mean, (S.D.)]	45.50	12.78	49.64	12.84	42.04	11.85	40.64	10.78
^§^Ethnicity (n,%)								
Black African	807.00	60.22	396.00	57.47	144.00	65.16	267.00	62.09
Other	533.00	39.78	293.00	42.53	77.00	34.84	163.00	37.91
Relationship status (n,%)								
Widowed/divorced/single/separated/ other	818.00	61.04	395.00	57.33	140.00	63.35	283.00	65.81
Married/living with partner	522.00	38.96	294.00	42.67	81.00	36.65	147.00	34.19
Site location (n,%)								
Urban	855.00	63.81	472.00	68.51	107.00	48.42	276.00	64.19
Rural	485.00	36.19	217.00	31.49	114.00	51.58	154.00	35.81
**Socio-economic**								
Education (n,%)								
No formal education (primary school or less)	437.00	32.61	257.00	37.30	68.00	30.77	112.00	26.05
Some high school	736.00	54.93	351.00	50.94	114.00	51.58	271.00	63.02
School leavers certificate &/or tertiary started or completed	167.00	12.46	81.00	11.76	39.00	17.65	47.00	10.93
Employment status (n,%)								
Unemployed	733.00	54.70	348.00	50.51	106.00	47.96	279.00	64.88
Employed part/full time	422.00	31.49	204.00	29.61	91.00	41.18	127.00	29.53
Pensioner/student/other	185.00	13.81	137.00	19.88	24.00	10.86	24.00	5.58
Predicted patient monthly income (2017 USD) [Median, (IQR)]	108.07	94.88	113.95	84.65	108.76	127.98	96.05	84.60
Household members hungry(n,%)								
Never hungry	685.00	51.12	333.00	48.33	154.00	69.68	198.00	46.05
Sometimes/seldom hungry	566.00	42.24	306.00	44.41	61.00	27.60	199.00	46.28
Often hungry	89.00	6.64	50.00	7.26	6.00	2.71	33.00	7.67
**Health status and wellbeing**								
Chronic co-morbidities (n,%)								
HIV+ only	718.00	53.58	235.00	34.11	151.00	68.33	332.00	77.21
DM only	539.00	40.22	397.00	57.62	60.00	27.15	82.00	19.07
HIV+ & DM	83.00	6.19	57.00	8.27	10.00	4.52	16.00	3.72
HRQoL [mean, (S.D.)]	0.79	0.23	0.76	0.24	0.92	0.13	0.78	0.23
HIV/DM stigma (n,%)								
Never	1251.00	93.36	660.00	96.07	211.00	95.48	380.00	88.37
Rarely/sometimes/often	87.00	6.49	27.00	3.93	10.00	4.52	50.00	11.63

§ We include “ethnicity” in our tables and regressions because race based on ethnicity is a social construct which was used during the Apartheid era in South Africa to confer advantage on some and disadvantage on others. Its effects are still entrenched in society today influencing socio-economic and health outcomes. Race and socio-economics are thus intertwined. Current social correction post-Apartheid is also guided by race due to the entrenched inequities.

**Table 2:** Monthly healthcare utilisation and patient costs by mental health condition

	MIND (All) (n=1340)		Depression only (n=689)		AUD only (n=221)		Depression & AUD (n=430)		
**Utilisation of healthcare services**									
**Visit type**	**Mean**	**(S.E.)**	**Mean**	**(S.E.)**	**Mean**	**(S.E.)**	**Mean**	**(S.E.)**	***p-value***
***Study clinic visit for routine HIV/DM care***	***0.73***	*0.01*	***0.73***	*0.02*	***0.72***	*0.03*	***0.73***	*0.02*	*0.99*
**Ambulatory visits (other health services)**	***1.11***	*0.12*	***1.19***	*0.14*	***0.70***	*0.15*	***1.18***	*0.27*	***<0.05***
Study clinic not for HIV/DM care	0.58	0.09	0.48	0.08	0.46	0.12	0.80	0.26	0.25
Other public clinic	0.06	0.01	0.09	0.02	0.03	0.02	0.04	0.01	0.41
Private provider consultations (Dr, Trad, Faith, NGO)	0.40	0.06	0.57	0.11	0.11	0.08	0.29	0.06	**<0.05**
Hospital outpatient (ER/OPD)	0.06	0.01	0.06	0.01	0.10	0.05	0.04	0.01	0.33
***Total ambulatory visits***	***1.84***	*0.12*	***1.93***	*0.14*	***1.41***	*0.15*	***1.91***	*0.27*	0.09
***Hospital inpatient days***	***0.08***	*0.02*	***0.09***	*0.03*	***0.09***	*0.03*	***0.08***	*0.03*	*0.50*
**Patient costs (USD)**									
**Cost type**	**Mean**	**(S.E.)**	**Mean**	**(S.E.)**	**Mean**	**(S.E.)**	**Mean**	**(S.E.)**	***p-vaVm***
**Direct costs (OOPP)**									
***Study clinic visit for routine HIV/DM care***	***0.86***	*0.05*	***0.88***	*0.07*	***0.59***	*0.11*	***0.95***	*0.10*	***<0.05***
***Other healthcare services total***	***1.26***	*0.19*	***1.92***	*0.34*	***0.34***	*0.13*	***0.67***	*0.20*	***<0.05***
Other public clinic & hospital ER/OPD	0.15	0.03	0.19	0.05	0.08	0.04	0.13	0.04	0.19
Private provider consultations (Dr, Trad, Faith, NGO)	1.11	0.18	1.73	0.33	0.26	0.12	0.54	0.20	***<0.05***
***Hospital inpatient***	***0.03***	*0.01*	***0.03***	*0.01*	***0.01***	*0.01*	***0.03***	*0.01*	*0.31*
***Healthcare products total***	***1.71***	*0.24*	***2.66***	*0.43*	***0.53***	*0.21*	***0.79***	*0.20*	***<0.05***
Traditional & faith based medicines	0.20	0.08	0.30	0.15	0.11	0.07	0.10	0.09	0.33
Pharmacy medicines/vitamins	0.49	0.07	0.65	0.13	0.34	0.14	0.32	0.09	0.09
Special diets	1.02	0.20	1.72	0.37	0.09	0.09	0.37	0.16	***<0.05***
***Total direct costs (OOPP)***	***3.86***	*0.33*	***5.49***	*0.58*	***1.47***	*0.29*	***2.44***	*0.34*	***<0.05***
***Total direct costs (OOPP excl. special diets)***	***2.84***	*0.25*	***3.78***	*0.44*	***1.39***	*0.27*	***2.07***	*0.30*	***<0.05***
**Indirect costs**									
Opportunity costs of time	2.93	0.12	3.02	0.16	2.92	0.35	2.79	0.20	0.09
Income loss	1.54	0.28	1.27	0.38	1.59	0.45	1.93	0.56	0.12
***Total indirect costs***	***4.46***	*0.30*	***4.29***	*0.41*	***4.51***	*0.55*	***4.72***	*0.59*	*0.23*
***Total patient costs (USD)***	***8.32***	*0.46*	***9.78***	*0.72*	***5.98***	*0.64*	***7.16***	*0.77*	***<0.05***
***Total patient costs (USD)(excl. special diets)***	***7.30***	*0.40*	***8.07***	*0.61*	***5.90***	*0.64*	***6.79***	*0.69*	***<0.05***
**Spending (OOPP) on special diets by HIV or DM**	**MIND (All) (n=1340)**		**Depression only (n=689)**		**AUD only (n=221)**		**Depression & AUD (n=430)**		
***Special diets***	**Mean**	**(S.E.)**	**Mean**	**(S.E.)**	**Mean**	**(S.E.)**	**Mean**	**(S.E.)**	***p-value***
***Special diets***	***1.02***	*0.20*	***1.72***	*0.37*	***0.09***	*0.09*	***0.37***	*0.16*	***<0.05***
HIV ***(n*=718)**	0.43	0.18	1.02	0.54	0.13	0.13	0.15	0.08	***<0.05***
DM ***(n*=539)**	1.58	0.39	1.87	0.51	0.00	0.00	1.32	0.75	***<0.05***
HIV&DM **(*n*=83)**	2.41	1.08	3.52	1.56	0.00	0.00	0.00	0.00	***0.13***
***p-value***	***<0.05***		***<0.05***		***0.79***		***<0.05***		

**Table 3:** Multiple linear regressions exploring predictors of OOPP & total patient costs

	*Total OOPP*		*Total OOPP excluding* *special diets*		*Total patient costs*		*Total patient costs* *excluding special diets*	
Variables	Estimate	*p-value*	Estimate	*p-value*	Estimate	*p-value*	Estimate	p-value
Location (1=urban)	34.33**	0.01	19.03**	0.02	54.23**	0.01	38.93**	0.01
Age (reference=18-30)								
*31*−*39*	21.73	0.19	4.90	0.65	38.88**	0.04	22.04	0.16
*40*−*49*	−9.21	0.41	−9.81	0.37	0.68	0.96	0.09	0.99
*50 +*	−9.33	0.58	−8.17	0.54	−10.27	0.59	−9.11	0.59
Sex (1=female)	6.56	0.42	−1.04	0.89	−11.62	0.44	−19.23	0.22
Ethnicity (1=Black African)	11.96	0.24	12.59	0.09	2.79	0.86	3.42	0.81
Marital status (1=married/partner)	10.46	0.13	−3.16	0.56	9.53	0.36	−4.09	0.68
Education (1=completed high school)	40.52**	0.02	42.55**	0.01	23.29	0.30	25.32	0.23
Employment (reference=unemployed)								
Employed(full/part-time)	19.52	0.22	10.75	0.29	64.87***	0.00	56.10***	0.00
Student/pensioner	0.58	0.97	13.24	0.42	5.21	0.79	17.87	0.35
Binary wealth index (1=less poor)	9.02	0.30	2.99	0.65	7.04	0.56	1.01	0.92
Food insecure(1=never)	10.60	0.18	6.81	0.40	−11.62	0.40	−15.41	0.22
Housing (1=stable housing)	6.59	0.56	4.23	0.65	−1.51	0.92	−3.87	0.80
Chronic disease (reference =HIV+ only)								
DM only	21.99	0.13	8.00	0.58	26.57	0.18	12.58	0.51
HIV+ & DM	26.19	0.29	3.88	0.79	98.08**	0.02	75.76**	0.03
Depression								
CES-D total score	0.32	0.32	0.34	0.17	0.35	0.50	0.37	0.41
Alcohol use								
AUDIT total score	−1.06**	0.04	−0.58	0.06	−0.66	0.45	−0.18	0.79
Health Related Quality of Life (HRQoL)index	−36.95**	0.03	−33.65**	0.01	−38.48	0.24	−35.19	0.25
Ambulatory visits	4.40	0.15	3.95	0.16	4.57	0.17	4.12	0.18
Hospital visits	7.77	0.35	9.03	0.29	12.48	0.33	13.75	0.30
HIV/DM stigma(1=yes)	11.43	0.55	2.83	0.76	23.70	0.43	15.10	0.53
***p<0,01 **p<0,05								

**Table 4:** Logistic regressions exploring drivers of income loss & catastrophic costs

	Income Loss Index (1=yes)	
Variable	OR	*p-value*
Location (1=urban)	1.63	0.13
Age (reference=18-30)		
*31*−*39*	0.81	0.52
40−49	0.73	0.49
*50 +*	0.75	0.47
Sex (1=female)	0.77	0.31
Ethnicity (1= Black African)	0.50**	0.01
Marital status(1=married/partner)	0.73	0.24
Education (1=completed high school)	0.41	0.19
Employment (reference=unemployed)		
Employed(full/part-time)	29.38***	0.00
Pensioner/student	1.40	0.64
Binary wealth index (1=less poor)	0.43***	0.00
Food insecure(1=never)	0.80	0.41
Housing (1=stable housing)	0.95	0.87
Chronic disease (reference=HIV+ only)		
DM only	0.83	0.57
HIV+ & DM	1.85	0.29
Depression: binary index of CES-D score (1=high>=16)	1.03	0.92
Alcohol use: binary index of AUDIT score (1= high>=8)	1.29	0.41
Health Related Quality of Life (HRQoL)index	0.90	0.90
Ambulatory visits	1.04**	0.01
Hospital visits	1.01	0.94
HIV/DM stigma(1=yes)	1.07	0.87
***p<0,01 **p<0,05 OR=odds ratio	Pseudo R2= 0.2625	

	Catastrophic Cost Index (OOPP) (1=yes)				Catastrophe Cost Index (TC) (1=yes)			
	Costs incl. diet		Costs excl. diet		Costs incl. diet		Costs excl. diet	
Variable	OR	*p-value*	*OR*	*p-value*	OR	*p-value*	*OR*	*p-value*
Location (1=urban)	2.32	0.08	2.15	0.12	1.33	0.28	1.18	0.54
Age (reference=18-30)								
*31*−*39*	1.37	0.39	1.15	0.70	1.24	0.21	1.19	0.31
40−49	0.76	0.52	0.66	0.35	0.79	0.34	0.78	0.30
*50 +*	0.52	0.15	0.46	0.07	0.73	0.15	0.76	0.23
Sex (1=female)	0.97	0.94	0.83	0.61	1.17	0.40	1.08	0.69
Ethnicity (1=Black African)	1.83**	0.03	1.83	0.09	1.26	0.31	1.18	0.47
Marital status(1=married/partner)	0.82	0.25	0.56***	0.00	0.81	0.14	0.71**	0.01
Education (1=completed high school)	1.02	0.93	1.28	0.47	0.78	0.36	0.81	0.40
Employment (reference=unemployed)								
Employed(full/part-time)	0.81	0.50	0.67	0.23	0.89	0.56	0.88	0.55
Pensioner/student	0.65	0.28	1.00	0.99	0.44**	0.02	0.48**	0.04
Binary wealth index (1=less poor)	1.27	0.24	1.23	0.48	1.06	0.65	1.07	0.65
Food insecure(1=never)	1.26	0.22	1.27	0.34	0.69	0.05	0.66**	0.03
Housing (1=stable housing)	0.97	0.92	0.90	0.75	0.89	0.49	0.86	0.38
Chronic disease (reference=HIV+ only)								
DM only	1.87**	0.03	1.49	0.40	1.08	0.63	0.88	0.59
HIV+ & DM	1.49	0.32	1.07	0.86	1.92**	0.03	1.92**	0.03
Depression: binary index of CES-D score (1=high>=16)	1.13	0.76	1.02	0.95	0.73	0.22	0.73	0.24
Alcohol use: binary index of AUDIT score (1= high>=8)	0.48***	0.00	0.54**	0.02	0.68	0.05	0.80	0.33
Health Related Quality of Life (HRQoL)index	0.38**	0.01	0.38***	0.00	0.55	0.11	0.59	0.21
Ambulatory visits	1.04**	0.04	1.03	0.06	1.04	0.22	1.04	0.23
Hospital visits	1.21	0.09	1.26**	0.03	1.03	0.77	1.07	0.57
HIV/DM stigma(1=yes)	1.41	0.14	1.70**	0.04	1.51**	0.02	1.59***	0.00
***p<0,01 **p<0,05 OR=odds ratio	Pseudo R2= 0.0915		Pseudo R2= 0.0863		Pseudo R2= 0.0613		Pseudo R2= 0.0596	

## Data Availability

A data dictionary and de-identified participant data will be made available after approval of a proposal and a signed data access agreement. Requests can be made to bronwyn.myers-franchi@curtin.edu.au.

## Box 1 Analytical framework equation

OOP payments = f(SES, HRQoL, CESD, AUDIT, health service utilisation, 〖chronic disease〗 _(HIV/DM), stigma; age, gender, race, martial status, employment, education, food security, location)

TC(OOP payments + IC) = f (SES,HRQoL, CESD, AUDIT, health service utilisation, 〖chronic disease〗 _(H IV/DM), stigma; age, gender,race,marital status, employment, education, food security, location)

Key: OOP: Out-of-Pocket ; SES: Socio-Economic Status; HRQoL: Health Related Quality of Life; CES-D: Centre for Epidemiology Scale on Depression; AUDIT: Alcohol Use Disorder Identification Test; HIV: Human Immunodeficiency Virus; DM: Diabetes Mellitus; TC: Total Costs; IC: Indirect Costs

## Highlights

This study uses baseline data from participants enrolled in a cluster randomised controlled trial. We describe patient costs (out-of-pocket payments (OOPP) and indirect costs) related to the utilisation of services and identify factors associated with the economic burden of untreated co-occurring depression and/or alcohol use disorders (AUDs) among patients with HIV and/or diabetes using government health services in the Western Cape Province of South Africa. Highlights from the study are:

- Mean monthly total patient costs: depression ($9.78); AUD ($5.98), & AUD & depression ($7.16).
- Clients with depression reported more private healthcare visits & OOP payments than the AUD group.
- Total patient costs were positively associated with urban location, employment & HIV/DM illness.
- Higher utilisation was associated with greater odds of incurring income losses.
- Results indicate a concerning economic burden, with cost and time as barriers to accessing care.
